# Phenotypic Analysis of the Chicken Thymic Microenvironment During Ontogenic Development

**DOI:** 10.1155/1992/32341

**Published:** 1992

**Authors:** Trevor J. Wilson, Natalie J. Davidson, Richard L. Boyd, M. Eric Gershwin

**Affiliations:** 1 Department of Internal Medicine/Rheumatology School of Medicine TB 192 University of California Davis California 95616 USA; 2 Department of Pathology and Immunology Monash University Medical School Melbourne Australia

**Keywords:** Thymus, chicken, monoclonal antibodies, ontogeny

## Abstract

The development of monoclonal antibodies (mAb) reactive with the thymic microenvironment has identified distinct subpopulations within the stromal component, but the function of these subregions in intrathymic T-cell differentiation remains essentially an enigma. In this study, we have used such a panel of mAb to examine the chicken thymus during ontogenic development to gain insight into the contributions of these thymic regions to the distinct phases of T-cell development and to further characterize the development of this organ. Our reagents have demonstrated the complex differentiation of the primitive endodermal epithelium into more specialized structures and the development of other thymic stromal components from mesectodermal cells. We also describe molecules localized to the subcapsular and perivascular regions, which have an ontogenic expression corresponding to the early localization and stimulation of thymic precursors and another molecule on the medullary vasculature expressed corresponding to the exit of mature cells from the thymus. In addition, two markers of distinct medullary epithelial clusters are initially expressed corresponding to the appearance of T-cell receptor-1 (TcR-1) and TcR-2 positive cells in the medulla, respectively. These mAb potentially represent excellent reagents for further definition of the thymic modulation of T-cell differentiation.


*Developmental Immunology*, 1992, Vol. 2, pp. 19–27
Reprints available directly from the publisher
Photocopying permitted by license only

© 1992 Harwood Academic Publishers GmbH
Printed in the United Kingdom

### Phenotypic Analysis of the Chicken Thymic Microenvironment During Ontogenic Development

TREVOR J. WILSON,*† NATALIE J. DAVIDSON,‡ RICHARD L. BOYD,‡ and M. ERIC GERSHWIN†

†*Department of Internal Medicine/Rheumatology, School of Medicine TB 192, University of California, Davis, California 95616*
‡*Department of Pathology and Immunology, Monash University Medical School, Melbourne, Australia*

The development of monoclonal antibodies (mAb) reactive with the thymic microenvironment has identified distinct subpopulations within the stromal component, but the function of these subregions in intrathymic T-cell differentiation remains essentially an enigma. In this study, we have used such a panel of mAb to examine the chicken thymus during ontogenic development to gain insight into the contributions of these thymic regions to the distinct phases of T-cell development and to further characterize the development of this organ. Our reagents have demonstrated the complex differentiation of the primitive endodermal epithelium into more specialized structures and the development of other thymic stromal components from mesectodermal cells. We also describe molecules localized to the subcapsular and perivascular regions, which have an ontogenic expression corresponding to the early localization and stimulation of thymic precursors and another molecule on the medullary vasculature expressed corresponding to the exit of mature cells from the thymus. In addition, two markers of distinct medullary epithelial clusters are initially expressed corresponding to the appearance of T-cell receptor-1 (TcR-1) and TcR-2 positive cells in the medulla, respectively. These mAb potentially represent excellent reagents for further definition of the thymic modulation of T-cell differentiation.

KEYWORDS: Thymus, chicken, monoclonal antibodies, ontogeny.

### INTRODUCTION

The thymic microenvironment has been shown to be essential for the differentiation of mature, functional, TcR-expressing, major histocompatibility complex-restricted T cells. The mechanisms within the thymic microenvironment that control this process, however, remain relatively poorly understood. A recent approach to elucidating the specific function of each of the thymic nonlymphoid components (comprised essentially of epithelium, macrophages, dendritic cells, and connective tissue) has been the production of specific mAb. Panels of such mAb have been described for the chicken, human, mouse, and rat thymus (Haynes, 1984; van Vliet et al., 1984; Boyd et al., 1988; Godfrey et al., 1990). These reagents have defined specific regions within this organ, identified previously undescribed subpopulations of stromal cells, and emphasized the complex yet phylogenically conserved nature of the thymus. Most importantly, the phenotypic characterization of thymic structure has enabled the identification of stromal-cell molecules that potentially interact with various subpopulations of developing T cells (Kingston et al., 1984; van de Water et al., 1990; Boyd et al., 1991a).

The study of aves has greatly contributed to the understanding of embryogenesis (Le Douarin et al., 1975; Houssaint et al., 1976) and the dichotomy of the immune system (Glick et al., 1956). Chick-quail chimeras have been used to define the relative contributions of the endodermal epithelium, mesectodermal-derived mesenchyme, and bloodborne extrinsic elements to thymic histology (Le Douarin and Jotereau, 1975). Thus, we previously raised and characterized a panel of mAb reactive with the chicken thymus, which are described elsewhere (Boyd et al., 1991b). These reagents identify subpopulations of the thymic stroma including pan-epithelium, isolated corti-

*Corresponding author. Present address: Department of Internal Medicine/Rheumatology, School of Medicine TB 192, University of California, Davis, CA 95616.

TABLE 1
Ontogenic Distribution of mAb That Stain Adult Thymic Epithelium[a]

| Localization posthatching | mAb | E5 | E10–E12[b] | E16–E18[b] |
|---|---|---|---|---|
| Cortical and med Ep | MUI-54 | Pan-Ep | Pan-Ep | Pan-Ep |
| Isolated cortical Ep | MUI-52 | Negative | Negative | Isolated cortical Ep |
| Med Ep clusters | MUI-62 | Negative | Isolated Ep | Med Ep clusters |
| ST/SC, med PV Ep (type I) | MUI-70 | Negative | Isolated Ep | Isolated ST, Med PV Ep |
| Subpopulations of Type I | MUI-53 | Negative | Isolated Ep | Isolated ST, med PV Ep |
| Isolated ST/SC Ep and med Ep | MUI-58 | Negative | Isolated Ep | Med Ep |

[a]Abbreviations: Ep, epithelial; med, medulla; PV, perivascular; ST, subtrabecular; SC, subcapsular.
[b]Although multiple ages were examined, results are grouped because no significant difference was observed between these ages.

cal epithelium, medullary epithelial clusters, and subcapsular, subtrabecular, and medullary perivascular epithelium. In this study, we have further defined the histogenesis of the thymus and have identified molecules that may potentially have interactive roles with differentiating T cells.

### RESULTS

### MAb That Identify Thymic Epithelial Cells (Table 1)

MUI-54 stained the entire epithelium throughout ontogeny (Figs. 1(a) and 1(b)) until 3 days after hatching when the antigen was detected on cortical and medullary epithelium, but only on restricted regions of the subtrabecular and subcapsular epithelium. The subtrabecular, subcapsular, and perivascular epithelium (Type I; van de Wijngaert et al., 1984) were identified by MUI-70 (Figs. 1(c) and 1(d)) and subpopulations of this region were identified by MUI-53. The mAb MUI-53 and -70 were negative on the thymic primordium at E5 (days of embryogenesis) and were initially detected at E10 on a subpopulation of keratin-positive cells (Fig. 1). From E14, these cells were localized in perivascular areas of the developing medulla. During ontogeny, trabeculae were observed slowly extending into the cortex. At approximately E16–E18, these connective tissue septae reached the medulla and isolated regions of subtrabecular epithelium were observed to express antigens defined by MUI-53 and -70. Posthatching, these markers were expressed as observed in the adult. MUI-58 labeled adult medullary epithelium and a subpopulation of subtrabecular and subcapsular epithelium. This molecule was initially observed on isolated, centrally located epithelial cells in E10 lobes, medullary epithelial cells throughout ontogeny, and on the subtrabecular epithelium from E20.

MUI-62 stained adult thymic medullary epithelial clusters and fine granules surrounding these cells. Although the MUI-62-defined antigen was not expressed on the E5 thymic primordium and rarely expressed on the E10 thymus, this marker was consistently observed on isolated, centrally located epithelial cells at E12 (Figs. 2(a) and 2(b)). By E14, these positive epithelial cells had formed the medullary clusters that were observed throughout further development. Isolated MUI-52 positive epithelial cells were located in the adult cortex. These MUI-52-expressing cells were initially observed in the E14 thymus and subsequently located in the cortex throughout ontogenic development.

### MAb That Identify Thymic Epithelial and Non-Epithelial Cells (Table 2)

The mAb MUI-72 and -80 labeled adult thymic epithelial medullary clusters though their antigens were also detected on keratin-negative reticular fibroblastlike and macrophagelike cells, respectively, scattered throughout the cortex and

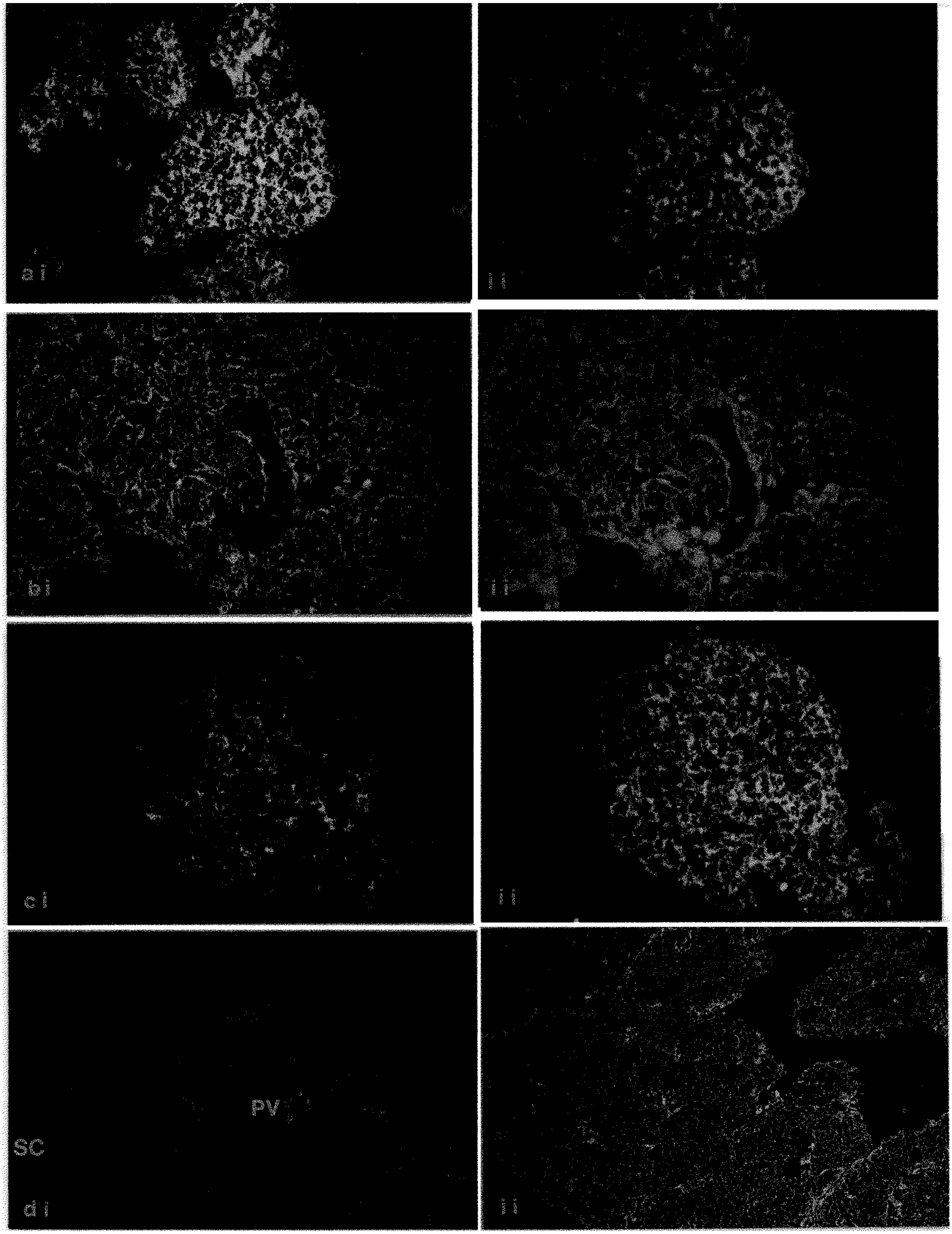

FIGURE 1. MUI-54 staining of E10 (a) and E20 thymus (b) (×250). Note labeling of all thymic epithelium. MUI-70 staining of isolated epithelium in E10 thymic lobe (c; ×250) and subcapsular (SC) and perivascular labeling (PV) of adult thymus (d; ×125). The first of each pair of photographs shows mAb labeling (i) and the second shows double labeling with antikeratin (ii).

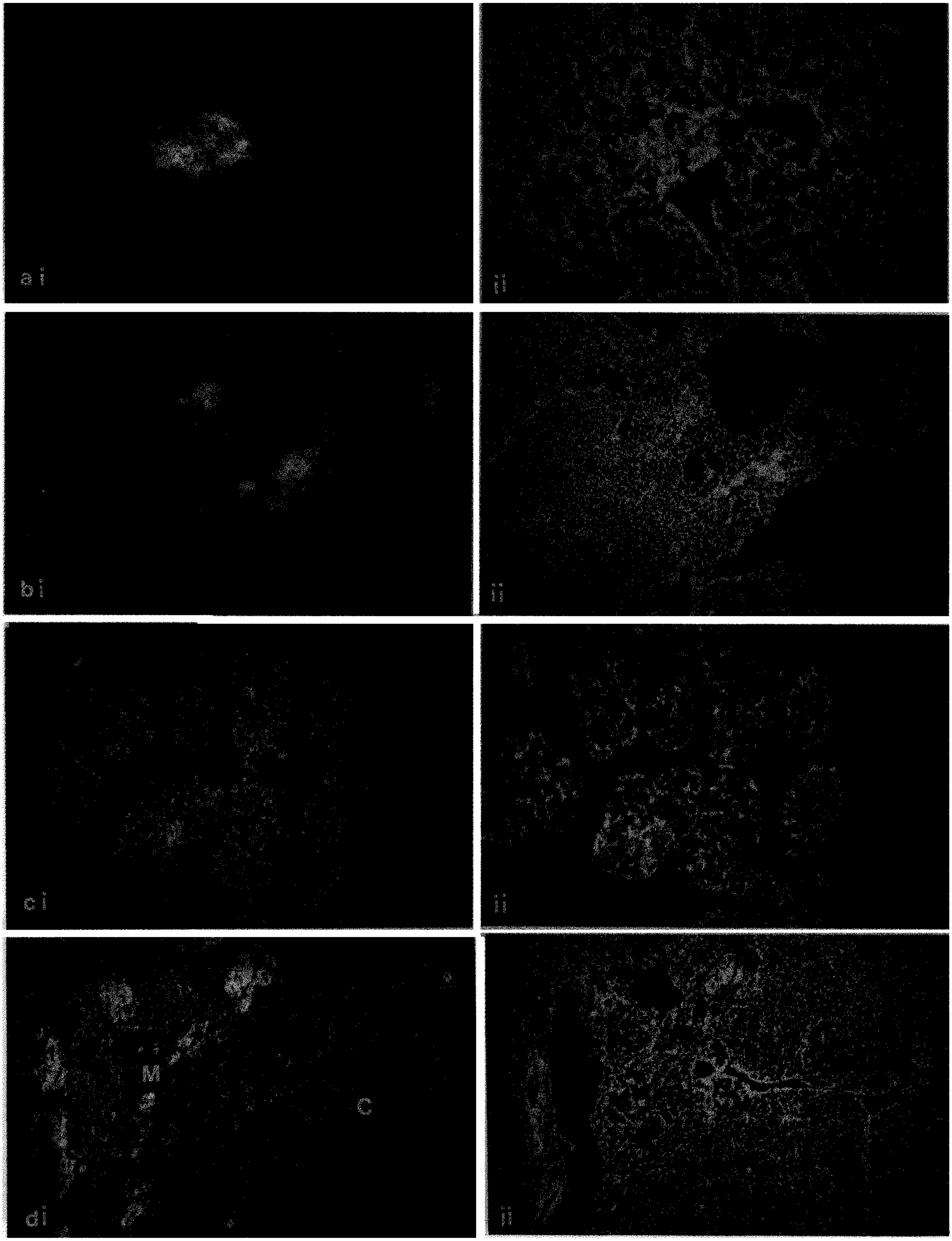

FIGURE 2. MUI-62 staining of isolated epithelium in E14 thymus (a; ×250) and medullary epithelial clusters in the adult (b; × 125) thymus. MUI-66 staining of entire E10 thymic epithelium (c; ×250) and isolated kerain-positive and negative cells together with medullary epithelial clusters in the adult thymus (d; ×125). Medulla (M) and cortex (C) are indicated. The first of each pair of photographs shows mAb labeling (i) and the second shows double labeling with antikeratin (ii).

TABLE 2
Ontogenic Distribution of mAb That Label Adult Thymic Epithelial and Non-Epithelial Cells[a]

| Localization posthatching | mAb | E5 | E10–E12[b] | E16–E18[b] |
|---|---|---|---|---|
| Isolated cortex Ep, confluent med Ep and non-Ep | MUI-78 (B-L) | Negative | Isolated Ep and non-Ep | Isolated cortical Ep, confluent med |
| Isolated $K^{+/-}$ cells and med Ep clusters | MUI-72 | Negative | Isolated Ep | Med Ep clusters |
| Isolated $K^{+/-}$ cells and med Ep clusters | MUI-80 | Negative | Isolated Ep | $K^{+/-}$ cells and med Ep clusters |
| Isolated $K^{+/-}$ cells and med Ep clusters | MUI-66 | Pan-Ep | Pan-Ep | Isolated $K^{+/-}$ cells and med Ep clusters |

[a]Abbreviations: Ep, epithelial; K, keratin; med, medulla.
[b]Although multiple ages were examined, results are grouped because no significant difference was observed between these ages.

medulla. MUI-80 was negative at E5 and initially positive on isolated epithelial cells at E10. By E14, MUI-80 had the same distribution as that observed in the adult. The MUI-72-defined antigen was first observed on centrally located epithelial cells at E12 and on the scattered reticular fibroblastlike cells from E20. Similarly, the MUI-66-defined marker was expressed on epithelial clusters in the medulla and scattered keratin-positive and negative cells throughout the cortex and medulla, including epithelial, reticular fibroblastlike, and macrophagelike cells (Figs. 2(c) and 2(d)). This marker labeled all the thymic epithelium at E5, E10, and E12 and became more restricted from E14, when it was expressed as observed in the adult. MUI-78 recognized a monomorphic determinant of the chicken MHC Class II molecule (B-L) on isolated stellate epithelial cells in the cortex and confluent medullary epithelial and non-epithelial cells. This molecule was initially detected on isolated epithelial and non-epithelial cells in the E10 thymus and had a

TABLE 3
Ontogenic Distribution of mAb That Label Adult Thymic Non-Epithelial Cells[a]

| Localization posthatching | mAb | E5 | E10–E12[b] | E16–E18[b] |
|---|---|---|---|---|
| Isolated med, ly, and MØ | MUI-36 | Negative | Rare isolated ly, and MØ | Isolated med ly, and MØ |
| Isolated MØ, especially med | MUI-79 | Negative | Rare isolated non-Ep cells | Isolated MØ, especially med |
| Med blood vessels | MUI-82 | Negative | Negative | Med blood vessels |
| Cortical and majority med ly | MUI-83 | Isolated cells | Rare isolated ly (10), confluent ly (12) | Confluent ly |
| Ly cortex and med | CD3 | Negative | Ly in $K^+$ areas | Ly cortex and med |
| Cortex: ly; med: isolated ly, more numerous in $K^-$ areas | CD4 | Negative | Negative | Ly cortex, isolated med |
| Cortex: ly; med: isolated ly, more numerous in $K^+$ areas | CD8 | Negative | Isolated ly in $K^+$ areas | Ly cortex, isolated med |
| Isolated med, especially $K^+$ areas | TcR-1 | Negative | Negative | Isolated med |

[a]Abbreviations: Ep, epithelial; K, keratin; ly, lymphocytes; med, medulla; MØ, macrophage.
[b]Although multiple ages were examined, results are grouped because no significant difference was observed between these ages.

distribution similar to that observed in the adult by E16.

### MAb That Identify Thymic Non-Epithelial Cells (Table 3)

MUI-79 stained a subpopulation of macrophages that was scattered throughout the thymus, although more concentrated in the medulla. MUI-79 was initially detected on isolated thymic non-epithelial cells in the E10 that were located as observed in the adult from E16. MUI-82, which reacted with blood vessels in the medulla of the adult thymus, was not observed in the thymus until E18. At this stage, it labeled rare medullary blood vessels, which became more numerous during development.

MUI-83 reacts strongly with cortical and more weakly with the majority of medullary thymocytes on tissue sections. Two-color flow cytometric analysis revealed that MUI-83$^+$ reacted with >95% of thymocytes; bearing CD3, CD4, and CD8, encompassing all subsets defined by CD3, CD4, and CD8 (Bean et al., in preparation). The MUI-83 marker was detected on rare isolated cells in the E5 thymic primordium and labeled rare lymphocytes in the E10 thymus (16% by FACS). The majority of thymocytes were positive for this marker at E12 (73%). MUI-36 identified a subpopulation of medullary lymphoid cells and macrophages in the mature thymus. In the embryonic thymus, isolated positive cells were observed from E10, although it was unclear whether these cells were lymphocytic or macrophages.

Significant numbers of cells expressing chicken TcR-1 and CD4 were not detected in sections until E14; the latter were detected mainly in the cortex and isolated TcR1$^+$ cells were observed in the medulla. CD8$^+$ cells were distributed similarly to CD4 in the E14 thymus, but isolated cells were observed in the thymic lobes at E10 and E12. The chicken CD3 marker was extensively expressed in the adult thymus labeling cortical and medullary lymphocytes. In the embryo, this marker was also present on many thymocytes from E10.

### DISCUSSION

We have utilized a panel of mAb to analyze the ontogenic development of the avian thymus, particularly, the development of specific stromal-cell types and the ontogenic expression of their phenotypic markers. These data have given important insights into the potential functions of these distinct molecules and the cells expressing them and have indicated that the differentiation events encompassing thymic histogenesis are as complex as the T-cell differentiation that the mature thymus regulates.

Le Douarin and Joterau (1975) demonstrated that the majority of thymic epithelium was derived from the endoderm. The mAb MUI-54 labeled a panthymic epithelium marker distinct from cytokeratin (Boyd et al., 1991b) throughout ontogeny, thus, during this phase, it may be specific for endodermal tissue. From 3 days post-hatching, however, this mAb displayed only limited reactivity to type I epithelium, indicating the more specialized nature of this region and demonstrating the differentiation of the endodermal epithelium. This was also indicated by MUI-66, which initially labeled all endodermal epithelium in the E5–E12 thymus, but in the adult, only stained medullary epithelial clusters, in addition to isolated epithelial and non-epithelial cells scattered throughout the cortex and medulla. This marker was also detected on areas of immature epithelium in the skin and gut (Boyd et al., 1991b) and on the bursal epithelium early in ontogenic development (Wilson and Boyd, 1990). Thus, this marker appears to be restricted to undifferentiated epithelial cells, perhaps precursors for more specialized epithelial subsets, supporting the concept of an epithelial cell precursor (Lampert and Ritter, 1988). The only other markers expressed on the E5 thymic primordium was detected by MUI-83, which recognizes an early T-cell marker (Bean et al., in preparation).

MUI-72 and -80 also recognized antigens on medullary epithelial clusters and isolated cells scattered in the cortex and medulla, but only detected isolated epithelial cells in the E10 thymus and were negative at E5. These cells were possibly of mesectodermal origin because isolated mesectodermal cells have been shown to infiltrate the endoderm prior to thymic histogenesis (Le Douarin and Jotereau, 1975). The ontogenic expression of these antigens supports the hypothesis that an interaction between the thymic endodermal and mesectodermal epithelium is required for normal thymic development (Auerbach, 1960). Similar interactions could also explain the common expression of many molecules on both thymic epithelial and non-epithelial cells (e.g., MHC class II; van Ewijk et al., 1980), although it is not known whether such markers were all endogenously synthesized, or perhaps some were passively acquired from the surrounding milieu.

The antigens expressed on medullary epithelial clusters or non-epithelial cells potentially may have a specific role in later stages of intrathymic T-cell development (e.g., acquisition of migratory capacity), because developing thymocytes are believed to undergo both positive and negative selection prior to entry into the medulla. They are unlikely to be involved in down regulation of CD4 or CD8 molecules because this has already begin in $CD4^{+}CD8^{+}CD3^{Hi}$ cells (Hugo et al., 1991). The stage of initial expression of the MUI-80- and -72-defined antigens on medullary clusters during ontogeny corresponds to the appearance in the medulla of TcR-1 and TcR-2 expressing cells, respectively (Chen et al., 1988; Coltey et al., 1989), although the significance of this expression to the development and migration of the TcR-1 and TcR-2 expressing thymocytes requires further examination.

MUI-62 defines another medullary epithelial marker, recognizing structures that may be the chicken equivalent of Hassall's corpuscles. This mAb also appeared to react with a ligand produced by these thymic epithelial cells and the endodermally derived epithelial lining of the gut (Bean et al., in preparation). This antigen was initially observed in the developing thymic medulla at E10–E12 and thus is probably expressed as a result of the specific differentiation of the thymic epithelium. MUI-52 defines another epithelial antigen that was initially observed relatively late in ontogeny, being first detected on isolated cortical cells at E14. This antigen was extensively expressed on thymic stromal cells in both heterogeneous cell culture and embryonic organ culture (Davidson et al., in preparation). This mAb, therefore, may reflect an activated state of stromal cells or possibly recognize a modulated antigen, for example, a growth factor receptor switched on *in vitro*.

In the adult, precursor cells have been shown to localize in the subcapsular cortex (Joterau and Le Douarin, 1982; Huiskamp and van Ewijk, 1985), which is the first region of the thymus to regenerate after sublethal irradiation (Huiskamp and von Ewijk, 1985; Huiskamp et al., 1985). It is also possible that type I epithelium may provide the stimulus for subcapsular lymphopoiesis, because thymic epithelium has been shown to induce precursor activation (Denning et al., 1988) and blasts are present in the subcapsule (Weissman, 1973). The late appearance of this phenotypically defined region defined by the mAb MUI-53 and -70 from approximately E16–E18 corresponds with the replacement of chick outer cortical thymocytes with quail precursor cells in the region in chick-quail chimeras (Coltey et al., 1989), as the third wave of precursor cells infiltrate the thymus (Le Douarine et al., 1984). This may reflect a role of the type I epithelium and possibly the MUI-53 and -70-defined molecules in the localization and/or stimulation of the precursors entering the thymus or repopulating the thymus after irradiation.

Similarly, the mAb MUI-58 was reactive with the thymic subcapsular and subtrabecular epithelium, but this antigen was also expressed on medullary epithelium (Boyd et al., 1991b). MUI-58 labeled isolated medullary epithelium in the E10 thymus, possibly of mesectodermal origin because neural crest mesectoderm has been shown to infiltrate the branchial arches prior to thymic histogenesis (Le Douarin and Jotereau, 1975) and is believed to generate the neuroendocrine epithelial components of the thymus (Haynes et al., 1983). This phylogenically conserved subcapsular/medullary antigen distribution has also been described in humans, mice, and rats (Haynes et al., 1984; Colic et al., 1988; Kampinga et al., 1989; Godfrey et al., 1990; Izon and Boyd, 1990) and is similar to the reactivity of A2B5, an mAb reactive with GQ gangliosides characteristic of neuroendocrine cells (Haynes et al., 1983). Hence, this mAb may react with mesectodermally derived neuroendocrine cells that are producing thymic factors or hormones. This is a particularly important mAb because it also has very restricted nonthymic reactivity (Boyd et al., 1991b).

Surface CD3, TcR-2 expressing T cells begin to seed the periphery from E19 (Coltey et al., 1989; Bucy et al., 1990), CD3 positive cells being <1% of splenocytes until hatching (Chen et al., 1986). The expression of the MUI-82 antigen on medullary blood vessels within the chicken thymus from E18 thus corresponds with the exit of these mature T cells, perhaps indicating the maturation of the vascular endothelium that may facilitate this migration.

The ontogenic analysis of this panel of mAb versus the avian thymus has demonstrated the differentiation of endodermal and mesectodermal thymic primordium into the specific functional components of the thymic microenvironment. Comparison of T-cell localization with the development of stromal antigens has revealed molecules that have an expression corresponding to the localization and stimulation of precursors in the thymic subcapsular region, the appearance of medullary TcR-1$^+$ and TcR-2$^+$ cells, and the exit of mature T cells from the thymus. The significance of the thymic nonlymphoid component requires much further examination, although the use of mAb in this ontogenic study has enabled potentially important molecules to be identified for further detailed analysis.

### MATERIALS AND METHODS

### Chickens

Australorp×White Leghorn F1 hybrid chicken embryos or fertile eggs were obtained from Research Poultry Farm (Research, Victoria) and incubated in a humidified incubator at 39 °C until required.

### Monoclonal Antibodies

MAb reactive with chicken thymic nonlymphoid tissue were produced in our laboratory and have been described in detail elsewhere (Boyd et al., 1991b). MAb reactive with chicken CD3, CD4, CD8, and TcR-1($\gamma\delta$-like) were kindly provided by Drs. C.-L. Chen and M.D. Cooper (Chen et al., 1986; Chan et al., 1988; Sowder et al., 1988).

### Tissue Sections

Chick embryos were sacrificed (≥6 per group) at 5, 10, 12, 14, 16, 18, and 20 days of embryogenesis, and chickens were killed immediately post-hatching and at 3, 7, 14, 28, and 42 days. Bursa, spleen, and thymus were removed, immersed in Tissue-Tek embedding medium (Miles Scientific), and snap-frozen on a liquid nitrogen-isopentane slurry. Cryostat sections (4 $\mu$m) were air dried onto glass slides and stained using standard indirect immunofluoresence procedures. An affinity-purified sheep antimouse immunoglobulin-FITC conjugate (1:100, Silenus Laboratories, Melbourne, Australia) was used to reveal first step mAb. Double labeling was performed using rabbit antikeratin (wide spectrum; 1:200; Dakopatts, Santa Barbara, California) and sheep antirabbit immunoglobulin-rhodomine conjugate (1:50; Silenus). Sections were examined using a Zeiss Axioskop microscope and a Zeiss MC100 camera and Kodak Ektachrome P800/1600 film were used for photography.

### ACKNOWLEDGMENTS

The authors wish to thank Dr. Claude Martin for assistance in the isolation of the E5 thymic primordium. This work was supported by grants from the National Institute of Health (Grant AR 36867), Medical Research Council, Australia (Grant No. 860396), and the Anticancer Council of Victoria (Grant No. 131658).

*(Received April 3, 1991)*

*(Accepted July 12, 1991)*

### REFERENCES

Auerbach R. (1960). Morphogenetic interactions in the development of the mouse thymus gland. Dev. Biol. **2:** 271–284.

Boyd R.L., Izon D.J., Godfrey D.I., Wilson T.J., Ward H.A., and Tucek C.L. (1988). Complex heterogeneity of the thymic stroma. Adv. Exp. Med. Biol. **237:** 263–268.

Boyd R.L., Wilson T.J., Van de Water J., Haapanen L.A., and Gershwin M.E. (1991a). Selective abnormalities in the thymic microenvironment associated with avian scleroderma, an inherited fibrotic disease of L200 chickens. J. Autoimmunity. **4:** 369–380.

Boyd R.L., Wilson T.J., Ward H.A. and Gershwin M.E. (1991b). Phenotypic characterization of chicken thymic stromal elements. Dev. Immunol. (in press).

Bucy R.P., Chen C.-L.H., and Cooper M.D. (1990). Ontogeny of T cell receptors in the chicken thymus. J. Immunol. **144:** 1161–1168.

Chan M.M., Chen C.H., Ager L.L., and Cooper M.D. (1988). Identification of the avian homologues of mammalian CD4 and CD8 antigens. J. Immunol. **140:** 2133–2138.

Chen C.H., Ager L.L., Gartland G.L., and Cooper M.D. (1986). Identification of a T3/T cell receptor complex in chickens. J. Exp. Med. **164:** 375–380.

Chen C.-L.H., Cihak J., Losch U., and Cooper M.D. (1988). Differential expression of two T cell receptors, TCR1 and TCR2, on chicken lymphocytes. Eur. J. Immunol. **18:** 539–543.

Colic M., Matanovic L., Hegedis L., and Dujic A. (1988). Immunohistochemical characterization of rat thymic nonlymphoid cells. I. Epithelial and mesenchymal components defined by monoclonal antibodies. Immunol. **65:** 277–284.

Coltey M., Bucy R.P., Chen C.H., Cihak J., Losch U., Char D.,

Le Douarin N.M., and Cooper M.D. (1989). Analysis of the first two waves of thymus homing stem cells and their progeny in chick-quail chimeras. J. Exp. Med. **170:** 543–557.

Denning S.M., Kirtzberg J., Le P.T., Tuck D.I., Singer K.H., and Haynes B.F. (1988). Human thymic epithelial cells directly induce activation of autologous immature thymocytes. Proc. Natl. Acad. Sci. USA **85:** 3125–3129.

Glick B., Chang T.S., and Jaap R.G. (1956). The bursa of Fabricius and antibody production. Poult. Sci. **35,** 224–245.

Godfrey D.I., Izon D.J., Tucek C.L., Wilson T.J., and Boyd R.L. (1990). Phenotypic heterogeneity of mouse thymic stromal cells. Immunology **70:** 66–74.

Haynes B.F. (1984). The human thymic microenvironment. Adv. Immunol. **36:** 87–142.

Haynes B.F., Scearce R.M., Lobach D.F., and Hensley L.L. (1984). Phenotypic characterization and ontogeny of mesodermal derived and endocrine components of the human thymic miocroenvironments. J. Exp. Med. **159:** 1149–1168.

Haynes B.F., Shimizu K., and Eisenbarth G.S. (1983). Identification of human and rat thymic epithelium using tetanus toxin and monoclonal antibody A2B5. J. Clin. Invest. **71:** 9–14.

Houssaint E., Belo M., and Le Douarin N.M. (1976). Investigations on cell lineage and tissue interactions in the developing bursa of Fabricius through interspecific chimeras. Dev. Biol. **53:** 250–264.

Hugo P., Boyd R.L., Waanders G.A., Petrie H.T., and Scollay R. (1991). Timing of deletion of autoreactive $V\beta6^+$ cells and down modulation of either CD4 or CD8 on phenotypically distinct $CD4^+CD8^+$ subsets of thymocytes expressing intermediate or high levels of T cell receptor. Internat. Immunol. **3:** 265–272.

Huiskamp R. and van Ewijk W. (1985). Repopulation of the mouse thymus after sublethal fission neutron irradiation. I. Sequential appearance of thymocyte subpopulations. J. Immunol. **134:** 2161–2169.

Huiskamp R., van Vliet E., and van Ewijk W. (1985). Repopulation of the mouse thymus after sublethal fission neutron irradiation. II. Sequential changes in the thymic microenvironment. J. Immunol. **134:** 2170–2178.

Izon D.J., and Boyd R.L. (1990). The thymus cytoarchitecture detected by monoclonal antibodies. Human Immunol. **27:** 16–32.

Jotereau F.V., and Le Douarin N.M. (1982). Demonstration of a cyclic renewal of the lymphocyte precursor cells in the quail thymus during embryonic and perinatal life. J. Immunol. **129:** 1869–1877.

Kampinga J., Berges S., Boyd R., Brekelmans P., Colic M., van Ewijk W., Kendall M.D., Ladyman H., Ritter M., Schuurman H.-J., and Tournefier A. (1989). Thymic epithelial antibodies: Immunohistological analysis and introduction of CTES nomenclature. Summary of the Roduc Epithelium Workshop. Thymus **13:** 165–173.

Kingston R., Jenkinson E.J., and Owen J.J.T. (1984). Characterization of stromal cell populations in the developing thymus of normal and nude mice. Eur. J. Immunol. **14:** 1052–1056.

Lampert I.A., and Ritter M.A. (1988). The origin of the diverse epithelial cells of the thymus: Is there a common stem cell? *In:* Thymus update. I. The microenvironment of the human thymus, Kendall M.D. and Ritter M.A., Eds. (Chur: Harwood Academic Publishers), pp. 5–25.

Le Douarin N.M., Dieterlen-Lievre F., and Oliver P.D. (1984). Ontogeny of primary lymphoid organs and lymphoid stem cells. Am. J. Anat. **170:** 261–299.

Le Douarin N.M., Houssaint E., Jotereau F.V., and Belo M. (1975). Origin of haemopoietic stem cells in embryonic bursa of Fabricius and bone marrow studied through interspecific chimeras. Proc. Natl. Acad. Sci. NY **72:** 2701–2705.

Le Douarin N.M., and Jotereau F.V. (1975). Tracing of cells of the avian thymus through embryonic life in interspecific chimeras. J. Exp. Med. **142:** 17–40.

Sowder J.T., Chen C.-L.H., Ager L.L., Chan M.M., and Cooper M.D. (1988). A large subpopulation of avian T cells express a homologue of the mammalian T $\gamma/\delta$ receptor. J. Exp. Med. **167:** 315–322.

van de Water J., Wilson T.J., Haapanen L.A., Boyd R.L., and Gershwin M.E. (1990). Ontogeny of T cell development in avian Scleroderma. Clin. Immunol. Immunopathol. **56:** 169–184.

van de Wijngaert F.P., Kendall M.D., Schuurman H.-J., Rademakers L.P.H.M., and Kater L. (1984). Heterogeneity of epithelial cells in the human thymus. An ultrastructural study. Cell. Tissue Res. **237:** 227–237.

van Ewijk W., Rouse R.V., and Weissman I.L. (1980). Immunoelectron microscopic identification of I-A and H-2k bearing cells. J. Histochem. Cytochem. **36:** 1084–1099.

van Vliet E., Melis M., and van Ewijk, W. (1984). Monoclonal antibodies to stromal cell types of the mouse thymus. Eur. J. Immunol. **14:** 524–529.

Weissman I.L. (1973). Thymus cell migration: Studies of the origin of cortisone resistant lymphocytes. J. Exp. Med. **137:** 504–510.

Wilson T.J., and Boyd R.L. (1990). The ontogeny of chicken bursal stromal cells defined by monoclonal antibodies. Dev. Immunol. **1:** 31–39.

